# Supplementary material for: Germline mutation landscape of DNA damage repair genes in African Americans with prostate cancer highlights potentially targetable RAD genes
Source: Nat Commun. 2022 Mar 15;13:1361. doi: 10.1038/s41467-022-28945-x (PMC8924169; doi:10.1038/s41467-022-28945-x)
Supplement: Supplementary file 1 — Supplementary Information [file 41467_2022_28945_MOESM1_ESM.pdf]

Supplementary Information

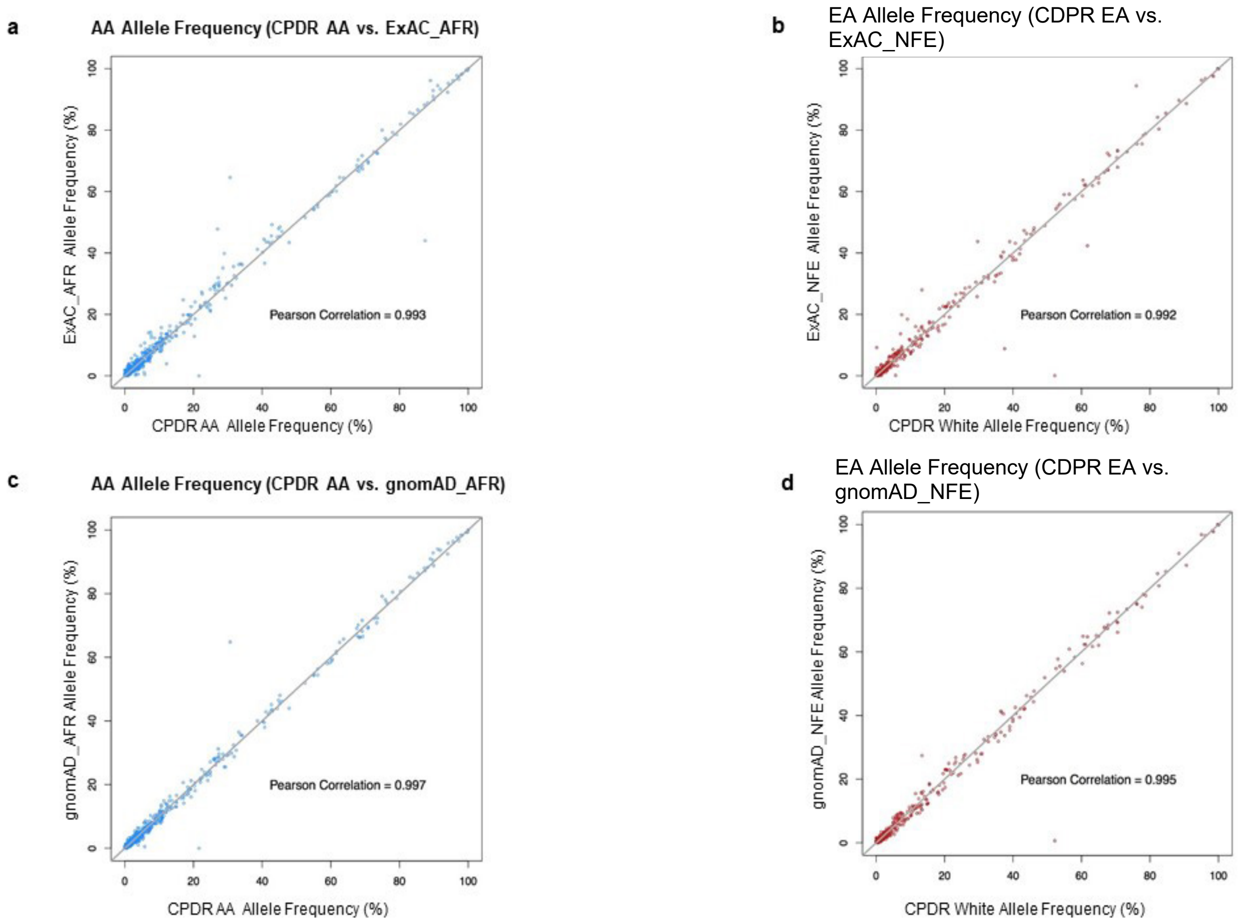

Supplementary Figure 1. Allele Frequency Comparison of Case and Control Populations.

Pearson correlation analysis were performed by comparing allele frequencies of 2,856 variants in DDRG regions in case cohort and corresponding healthy (control) populations in public databases (ExAC[1] and gnomAD[2]) at the same SNP site. Pearson correlations were above 0.99 in all four comparisons. A) AA cohort compared with ExAC AFR; B) EA cohort compared with ExAC NFE; C) AA cohort compared with gnomAD AFR; D) EA cohort compared with gnomAD NFE. Source data are provided as a Source Data file.

CPDR AA: African American samples in this study cohort; CPDR EA: EA American samples in this study cohort; ExAC AFR: African/African American population in ExAC database; ExAC NFE: Non-Finnish European population in ExAC database; gnomAD AFR: African/African American population in gnomAD database; gnomAD NFE: Non-Finnish European population in gnomAD database

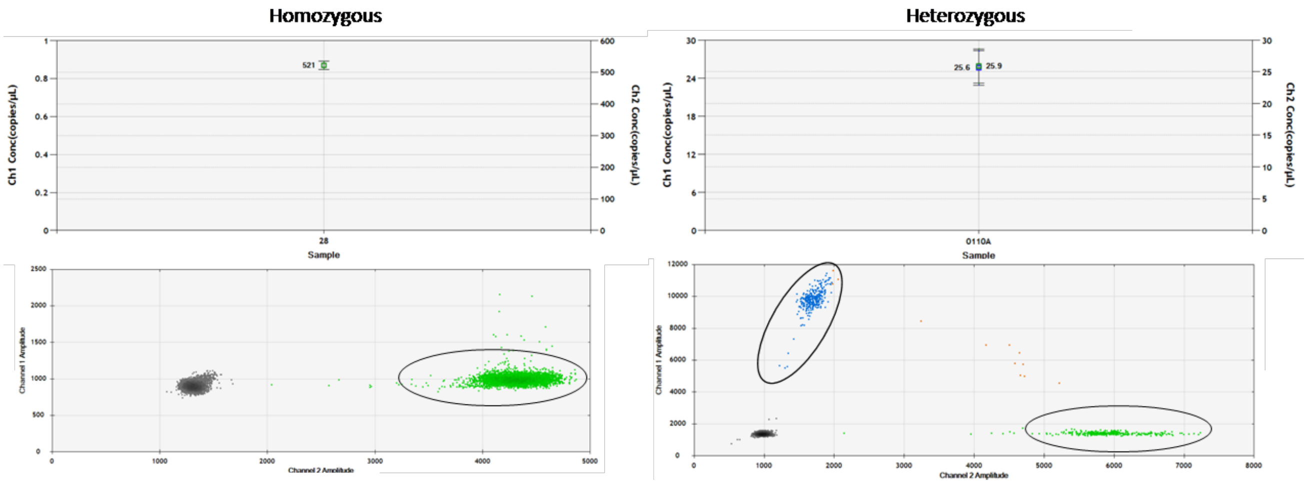

Supplementary Figure 2. SNV Genotyping (ddPCR) Analysis: Cluster Plot

Representative graph for SNP genotyping using droplet digital PCR (ddPCR) approach. Upper panel shows concentration (copies/ul) of FAM allele (G; Channel 1) and VIC allele (C; Channel 2) in set of representative samples with 2 genotypes (CC, GC). Lower panel is 2-D Amplitude view where each axis represents the amplitude of fluorescence for either FAM (vertical axis) or VIC (horizontal axis). The FAM probe can hybridize only to the alternate allele (G allele), while the VIC probe hybridizes only to reference allele (C allele). Source data are provided as a Source Data file.

Supplementary Table 1: Germline Mutation Frequency in *RAD* Gene Family across AA and EA Men

Gene	AA	EA
RAD51	7	0
RAD54L	6	2
RAD54B	4	1
RAD51C	1	0
Total	18	3

Gene	Germline mutation Carrier	Non-Carrier Controls	OR	P value
**AA**	18	241	6.68	0.0005
**EA**	3	269		

Two-sided Fisher Exact test was performed to assess the p-value; OR, Odds ratio; the gene names are italicized

Grouping all *RAD* mutations together we observed a greater mutation rate in AA (18/259; 6.95%) than in EA patients

Supplementary Table 2: **Pathway analysis based on 14 published known DDRG pathways**

Pathway	Total Germline Mutations	BCR p value (Logistic Regression)	BCR p value (Cox Hazard)	Metastasis p value (Logistic Regression)	Metastasis p value (Cox Hazard)
HR	15	0.072	0.018	0.623	0.431
NHEJ	3	0.373	0.488	0.045	0.053

P<0.05 corresponds to univariate association with BCR, Metastasis and the time to events; DDRG, DNA Damage Repair Gene; HR, Homologous Recombination; NHEJ, Non-Homologous End Joining; BCR, Biochemical Recurrence; Candidate pathway information is based on Knijnenburg *et al*, Cell Report 2018[3]

Supplementary Table 3: DDRG mutation status in targetable gene panel in AA and EA prostate cancer cases

Gene	AA	EA
FANCA	0	8
MSH6	1	3
FANCL	3	2
RAD54B	4	1
BRCA1	4	0
PMS2	5	0
RAD54L	6	2
RAD51	7	0
**Total**	30	16

DDRG Mutation Positive	DDRG Mutation Negative	OR	P value (95% CI)
30	229	2.1	0.021 (1.114 - 3.95)
16	256		

Two-sided Fisher Exact test was performed to assess the p-value; OR, Odds ratio; AA, African American; the gene names are italicized

Note: Only those genes are considered where mutation carrier frequency is at least 1%

Supplementary Table 4: Association of DDRG germline mutations with clinical outcomes in AA men

	No Mutations; N=200	Mutations (at least 1 or/and more mutations; N=59)	p value
**Gleason**			0.538
N-Miss	9	4	
3+3	95 (49.7%)	32 (58.2%)	
3+4	56 (29.3%)	13 (23.6%)	
4+3/8-10	40 (20.9%)	10 (18.2%)	
**Diagnosis Age**			0.313
old	152 (76.0%)	41 (69.5%)	
young	48 (24.0%)	18 (30.5%)	
**PSA Category**			0.214
1:<4	46 (23.0%)	13 (22.0%)	
2:4-9	128 (64.0%)	36 (61.0%)	
3:10-20	21 (10.5%)	5 (8.5%)	
4:>20	5 (2.5%)	5 (8.5%)	
**Clinical T Stage**			**0.021**
N-Miss	13	5	
T1a-T2a	176 (94.1%)	46 (85.2%)	
T2b-T2c	10 (5.3%)	5 (9.3%)	
T3a-T3c	1 (0.5%)	3 (5.6%)	
**Pathology T Stage**			0.764
N-Miss	18	11	
T1a-T2a	5 (2.7%)	1 (2.1%)	
T2b-T2c	133 (73.1%)	33 (68.8%)	
T3a-T3c	44 (24.2%)	14 (29.2%)	
**NCCN Risk**			**0.021**
N-Miss	20	4	
High	19 (10.6%)	13 (23.6%)	
Intermediate	64 (35.6%)	12 (21.8%)	
Low	97 (53.9%)	30 (54.5%)	
**BCR**			**0.032**
N-Miss	16	7	
0	163 (88.6%)	40 (76.9%)	
1	21 (11.4%)	12 (23.1%)	
**Metastasis**			0.442
no	194 (97.0%)	56 (94.9%)	
yes	6 (3.0%)	3 (5.1%)	

Two-sided chi-squared tests were performed to test differences between AA/EA. A Fisher's Exact test was used when 20% of cells <5 counts, DDRG, DNA Damage Repair Gene; PSA, Prostate Serum Antigen; NCCN, National Comprehensive Cancer Network; BCR, Biochemical Recurrence

Supplementary Table 5: Association of DDRG germline mutations with clinical outcomes in EA men

	No Mutations (N=206)	Mutations (at least 1 or/and more muations; N=66)	p value
**Gleason**			0.828
N-Miss	4	1	
3+3	123 (60.9%)	37 (56.9%)	
3+4	46 (22.8%)	17 (26.2%)	
4+3/8-10	33 (16.3%)	11 (16.9%)	
**Diagnosis Age**			0.891
old	167 (81.1%)	53 (80.3%)	
young	39 (18.9%)	13 (19.7%)	
**PSA Category**			0.243
1:<4	56 (27.2%)	25 (37.9%)	
2:4-9	132 (64.1%)	33 (50.0%)	
3:10-20	13 (6.3%)	6 (9.1%)	
4:>20	5 (2.4%)	2 (3.0%)	
**Clinical T Stage**			0.502
N-Miss	8	2	
T1a-T2a	176 (88.9%)	55 (85.9%)	
T2b-T2c	20 (10.1%)	9 (14.1%)	
T3a-T3c	2 (1.0%)	0 (0.0%)	
**Path T Stage**			0.959
N-Miss	11	7	
T1a-T2a	13 (6.7%)	4 (6.8%)	
T2b-T2c	121 (62.1%)	37 (62.7%)	
T3a-T3c	60 (30.8%)	18 (30.5%)	
T4	1 (0.5%)	0 (0.0%)	
**NCCN Risk**			0.208
N-Miss	20	3	
High	20 (10.8%)	4 (6.3%)	
Intermediate	48 (25.8%)	23 (36.5%)	
Low	118 (63.4%)	36 (57.1%)	
**BCR**			0.585
N-Miss	6	3	
No	169 (84.5%)	55 (87.3%)	
Yes	31 (15.5%)	8 (12.7%)	
**Metastasis**			0.575
No	197 (95.6%)	62 (93.9%)	
Yes	9 (4.4%)	4 (6.1%)	

Two-sided chi-squared tests were performed to test differences between AA/EA. A Fisher's Exact test was used when 20% of cells <5 counts, DDRG, DNA Damage Repair Gene; PSA, Prostate Serum Antigen; NCCN, National Comprehensive Cancer Network; BCR, Biochemical Recurrence

Supplementary Table 6: SNV Assays for ddPCR validation

Gene	Assay ID	Company
ATM	dHsaMDS836900447	Biorad
ATM	dHsaMDS637289153	Biorad
ATM	dHsaMDS487481422	Biorad
ATM	dHsaMDS376753397	Biorad
BRCA2	dHsaMDS186806869	Biorad
CHEK2	dHsaMDS322795459	Biorad
CHEK2	dHsaMDS612881353	Biorad
ERCC2	dHsaMDS980732312	Biorad
ERCC2	dHsaMDS654462608	Biorad
ERCC2	dHsaMDS192482848	Biorad
ERCC2	dHsaMDS653302778	Biorad
ERCC2	dHsaMDS521915998	Biorad
ERCC2	dHsaMDS524761873	Biorad
ERCC2	dHsaMDS428533855	Biorad
ERCC2	dHsaMDS315508923	Biorad
FAN1	dHsaMDS990437744	Biorad
FANCA	dHsaMDS436401567	Biorad
FANCC	dHsaMDS257370799	Biorad
FANCL	dHsaMDS351915441	Biorad
FANCL	dHsaMDS932208054	Biorad
MLH3	dHsaMDS683569425	Biorad
MSH6	dHsaMDS608435109	Biorad
MSH6	dHsaMDS775262304	Biorad
PMS2	dHsaMDS784051321	Biorad
PMS2	dHsaMDS189979053	Biorad
PMS2	dHsaMDS277411566	Biorad
POLG	dHsaMDS534712069	Biorad
POLG	dHsaMDS459658451	Biorad
POLG	dHsaMDS317083595	Biorad
RAD51	dHsaMDS770317426	Biorad
RAD54B	dHsaMDS521422112	Biorad
RAD54B	dHsaMDS185959583	Biorad
RAD54B	dHsaMDS527619288	Biorad

RAD54L	dHsaMDS206208032	Biorad
RAD54L	dHsaMDS396129615	Biorad
RAD54L	dHsaMDS983987358	Biorad
BRCA1	C_153130545_10	ThermoFisher Scientific
MUTYH	C__27860252_10	ThermoFisher Scientific
POLG	C_335909115_10	ThermoFisher Scientific
POLG	C_163508406_10	ThermoFisher Scientific
POLG	C_163475624_10	ThermoFisher Scientific
POLG	C_190154504_10	ThermoFisher Scientific
RAD51	C__33548791_10	ThermoFisher Scientific
RAD54B	C_173746145_10	ThermoFisher Scientific
RAD54L	C_166609719_10	ThermoFisher Scientific

Note: 2 pathogenic SNVs, rs80359212 (R3128X) in *BRCA2* and rs387906563 (G228fs) in *BRCA1* were also confirmed by CPDR- BRCA dataset, which is based on the targeted sequencing of *BRCA1* and *BRCA2*[20].

References for Supplementary Information

1. Karczewski, K.J. *et al.* The ExAC browser: displaying reference data information from over 60 000 exomes. *Nucleic Acids Res* 45, D840-D845 (2017).
2. Lek, M. *et al.* Analysis of protein-coding genetic variation in 60,706 humans. *Nature* 536, 285-91 (2016).
3. Knijnenburg, T.A. *et al.* Genomic and Molecular Landscape of DNA Damage Repair Deficiency across The Cancer Genome Atlas. *Cell Rep* 23, 239-254 e6 (2018).
4. Abida, W. et al. Prospective Genomic Profiling of Prostate Cancer Across Disease States Reveals Germline and Somatic Alterations That May Affect Clinical Decision Making. JCO Precis Oncol 2017(2017).
5. Robinson, D. et al. Integrative clinical genomics of advanced prostate cancer. Cell 161, 1215-1228 (2015).
6. Pritchard, C.C. et al. Inherited DNA-Repair Gene Mutations in Men with Metastatic Prostate Cancer. N Engl J Med 375, 443-53 (2016).
7. Castro, E. et al. PROREPAIR-B: A Prospective Cohort Study of the Impact of Germline DNA Repair Mutations on the Outcomes of Patients With Metastatic Castration-Resistant Prostate Cancer. J Clin Oncol 37, 490-503 (2019).
8. Nguyen-Dumont, T. et al. Rare Germline Pathogenic Variants Identified by Multigene Panel Testing and the Risk of Aggressive Prostate Cancer. Cancers (Basel) 13(2021).

9. Mijuskovic, M. et al. Rare germline variants in DNA repair genes and the angiogenesis pathway predispose prostate cancer patients to develop metastatic disease. Br J Cancer 119, 96-104 (2018).

10. Nicolosi, P. et al. Prevalence of Germline Variants in Prostate Cancer and Implications for Current Genetic Testing Guidelines. JAMA Oncol 5, 523-528 (2019).

11. Sartor, O., Yang, S., Ledet, E., Moses, M. & Nicolosi, P. Inherited DNA-repair gene mutations in African American men with prostate cancer. Oncotarget 11, 440-442 (2020).

12. Antonarakis, E.S. et al. Germline DNA-repair Gene Mutations and Outcomes in Men with Metastatic Castration-resistant Prostate Cancer Receiving First-line Abiraterone and Enzalutamide. Eur Urol 74, 218-225 (2018).

13. Annala, M. et al. Treatment Outcomes and Tumor Loss of Heterozygosity in Germline DNA Repair-deficient Prostate Cancer. Eur Urol 72, 34-42 (2017).

14. Na, R. et al. Germline Mutations in ATM and BRCA1/2 Distinguish Risk for Lethal and Indolent Prostate Cancer and are Associated with Early Age at Death. Eur Urol 71, 740-747 (2017).

15. Darst, B.F. et al. Germline Sequencing DNA Repair Genes in 5545 Men With Aggressive and Nonaggressive Prostate Cancer. J Natl Cancer Inst 113, 616-625 (2021).

16. Pomerantz, M.M. et al. The association between germline BRCA2 variants and sensitivity to platinum-based chemotherapy among men with metastatic prostate cancer. Cancer 123, 3532-3539 (2017).

17. Carter, H.B. et al. Germline Mutations in ATM and BRCA1/2 Are Associated with Grade Reclassification in Men on Active Surveillance for Prostate Cancer. Eur Urol 75, 743-749 (2019).

18. Agalliu, I. et al. Rare germline mutations in the BRCA2 gene are associated with early-onset prostate cancer. Br J Cancer 97, 826-31 (2007).

19. Momozawa, Y. et al. Germline Pathogenic Variants in 7636 Japanese Patients With Prostate Cancer and 12 366 Controls. J Natl Cancer Inst 112, 369-376 (2020).

20. Petrovics, G. et al. Increased frequency of germline BRCA2 mutations associates with prostate cancer metastasis in a racially diverse patient population. Prostate Cancer Prostatic Dis 22, 406-410 (2019).

21. Matejcic, M. et al. Pathogenic Variants in Cancer Predisposition Genes and Prostate Cancer Risk in Men of African Ancestry. JCO Precis Oncol 4, 32-43 (2020).
