# Supplementary material for: Germline mutation landscape of DNA damage repair genes in African Americans with prostate cancer highlights potentially targetable RAD genes
Source: Nat Commun. 2022 Mar 15;13:1361. doi: 10.1038/s41467-022-28945-x (PMC8924169; doi:10.1038/s41467-022-28945-x)
Supplement: Supplementary file 3 — Description of Additional Supplementary Files [file 41467_2022_28945_MOESM3_ESM.pdf]

Description of Additional Supplementary Files

File Name: Supplementary Data 1
Description: **Frequency of DDRG germline variants in patients with prostate cancer**

*includes-
Non-Hispanic white- 70 (M) 506 (MN); Non-Hispanic Black-4 (M) 36 (MN))
**The sample size were 561 (BAP1, BARD1, BRIP1, and FAM175A), 437 (GEN1), and 534 (CHEK2).
** Spanish Population
***Wood RD, Mitchell M, Lindahl T. Mutat Res. 2005 Sep 4; 577(1-2):275-83.
****The HR/FA pathway was the only pathway with a statisti-
cally significant association, with carriers of P/LP/D HR/FA
alleles having 1.27-fold increased risk of PCa death

File Name: Supplementary Data 2
Description: **DDRG germline mutation ddPCR validation summary**

42/43 mutations in 74 patients were validated by Droplet Digital PCR (ddPCR) method.
Total number of patients screened including controls (wt)- 119 (99.15% concordance, 118/119).
1893A sample was excluded due to QC

File Name: Supplementary Data 3
Description: **DDRG germline mutation functional scoring by Engenome eVai analysis**

Note: 98/100 variants were validated for the functional effect by Engenome eVai tool
