# Supplementary material for: Germline mutation landscape of DNA damage repair genes in African Americans with prostate cancer highlights potentially targetable RAD genes
Source: Nat Commun. 2022 Mar 15;13:1361. doi: 10.1038/s41467-022-28945-x (PMC8924169; doi:10.1038/s41467-022-28945-x)
Supplement: Supplementary file 7 — Reporting Summary [file 41467_2022_28945_MOESM7_ESM.pdf]

nature portfolio

Corresponding author(s): Indu Kohaar, Gyorgy Petrovics

Last updated by author(s): Dec 17, 2021

Reporting Summary

Nature Portfolio wishes to improve the reproducibility of the work that we publish. This form provides structure for consistency and transparency in reporting. For further information on Nature Portfolio policies, see our Editorial Policies and the Editorial Policy Checklist.

Statistics

For all statistical analyses, confirm that the following items are present in the figure legend, table legend, main text, or Methods section.

n/a	Confirmed	
☐	☒	The exact sample size (*n*) for each experimental group/condition, given as a discrete number and unit of measurement
☐	☒	A statement on whether measurements were taken from distinct samples or whether the same sample was measured repeatedly
☐	☒	The statistical test(s) used AND whether they are one- or two-sided<br>*Only common tests should be described solely by name; describe more complex techniques in the Methods section.*
☐	☒	A description of all covariates tested
☐	☒	A description of any assumptions or corrections, such as tests of normality and adjustment for multiple comparisons
☒	☐	A full description of the statistical parameters including central tendency (e.g. means) or other basic estimates (e.g. regression coefficient) AND variation (e.g. standard deviation) or associated estimates of uncertainty (e.g. confidence intervals)
☐	☒	For null hypothesis testing, the test statistic (e.g. *F*, *t*, *r*) with confidence intervals, effect sizes, degrees of freedom and *P* value noted<br>*Give P values as exact values whenever suitable.*
☒	☐	For Bayesian analysis, information on the choice of priors and Markov chain Monte Carlo settings
☒	☐	For hierarchical and complex designs, identification of the appropriate level for tests and full reporting of outcomes
☒	☐	Estimates of effect sizes (e.g. Cohen's *d*, Pearson's *r*), indicating how they were calculated

Our web collection on statistics for biologists contains articles on many of the points above.

Software and code

Policy information about availability of computer code

Data collection	Clinical data were collected using Oracle and MS Access databases
Data analysis	Illumina HiSeq Analysis Software (Isas, 6.19.1.403+NSv6): sequence processing and QC<br>bcl2fastq2.v2.20: Conversion of bcl files to fastq<br>Isaac Aligner (Isaac-04.17.06.15): Genome Alignment<br>Strelka2 Version 2.8.0: Germline Variant Calling<br>Illumina HiSeq Analysis Software 2.2: sequence processing and QC<br>Broad Picard 2.18: sequencing QC<br>ContEst (GATK3.6): Estimate cross-sample contamination<br>Peddy(v0.4.3): Ancestry detection and relatedness estimation<br>InterVar (v2.0.1): Variant interpretation using ACMG-AMP guideline<br>AnnoVar (2019): Variant annotation<br>R 3.5.0: Statistical analysis<br>bcftools (v1.9): VCF file manipulation<br>Integrated Variant Viewer (IGV v2.4): variant visualization<br>gvcfgenotyper (v2018.10.15): merge genome VCFs into cohort VCF<br>R 4.0.3: Statistical analysis,<br>eVai (version 2.2): functional scoring of the variants<br>QuantaSoft (v1.7.4.0917)

For manuscripts utilizing custom algorithms or software that are central to the research but not yet described in published literature, software must be made available to editors and reviewers. We strongly encourage code deposition in a community repository (e.g. GitHub). See the Nature Portfolio guidelines for submitting code & software for further information.

Data

Policy information about availability of data

All manuscripts must include a data availability statement. This statement should provide the following information, where applicable:

- Accession codes, unique identifiers, or web links for publicly available datasets
- A description of any restrictions on data availability
- For clinical datasets or third party data, please ensure that the statement adheres to our policy

The germline mutation summary statistics datasets that support the findings of this study are available in public dataset repository-
Kohaar, Indu; Petrovics, Gyorgy (2021): DDRG Germline Mutation Summary Statistics .xls. figshare. Dataset. https://doi.org/10.6084/m9.figshare.16682389.v1

Source data are provided with this paper.

The CPDR Multicenter National Database- It is a web-based centralized research data management system comprising of Oracle and MS based databases. Data can only be used under an approved IRB protocol.

1000 Genome database- https://www.internationalgenome.org/
Exome Aggregation Consortium (ExAC) database - http://exac.broadinstitute.org
gnomAD database - http://gnomad.broadinstitute.org/
AnnoVar databases: https://annovar.openbioinformatics.org/en/latest/user-guide/download/

Field-specific reporting

Please select the one below that is the best fit for your research. If you are not sure, read the appropriate sections before making your selection.

☒ Life sciences ☐ Behavioural & social sciences ☐ Ecological, evolutionary & environmental sciences

For a reference copy of the document with all sections, see nature.com/documents/nr-reporting-summary-flat.pdf

Life sciences study design

All studies must disclose on these points even when the disclosure is negative.

Sample size	According to Robinson et al (Cell, 2015) and Pritchard et al (NEJM, 2016), 8-12% of CA men with metastatic CaP have been identified with at least one germline DDRG mutations. This proportion significantly exceeds the prevalence of 5% among CAs with localized CaP. Previous data from CPDR showed that BRCA2, the most often mutated DDRG in CaP, has a significantly increased mutation frequency in AA men as compared to CA patients. We assumed that by oversampling on AA in the study cohort, the frequency of germline DRG mutations will increase to as high as 15-20% for metastatic CaP patients and it will also increase among localized CaP group (5-6%). Based on these values, a total sample size of 600 patients (CA=300 plus AA=300), to include all 50 (8.3%) patients with confirmed metastatic CaP, and 550 men with localized CaP (plus ≥10 years follow up), would allow us to achieve statistical power of 70-90%, using a two-sided test and a summary alpha level of 0.05.
Data exclusions	Patients who provided informed consent to both the Center for Prostate Disease Research (CPDR) Multi-Center National Database and Biospecimen databank at the Walter Reed National Military Medical Center (WRNMMC) are the source of patients for this study. Eligible patients include all men aged 18 and older who are recommended for biopsy for detection of prostate cancer. All patients are military health care beneficiaries and their dependents. Men who cannot provide informed consent were excluded.<br>In the present study, the final analysis was performed in 531 CaP patients, including 259 AA and 272 CA, after excluding 11.5% (69/600) of the patients because of low quality sequencing results due to DNA yields, fragment size and contamination (N=26), or mismatch between genomic ancestry and self-reported race (N=33)
Replication	The most frequent (over 1% carrier frequency) and potentially clinically targetable mutations including the novel ones were confirmed (experimentally validated) by the WGS-independent ddPCR method (Supplementary Table 2).<br>To determine population similarities between our cohort and reference control cohorts with common variants, we compared common variant allele frequencies (> 1%) and observed a high degree of correlation between AA prostate cancer and AA controls, as well as between CA prostate cancer and CA control individuals (r2>0.99), demonstrating that population structure was similar across cases and controls in each race group (Supplementary Figure 2). This data replicates the findings between cases and controls in each race group for common variants.
Randomization	We have oversampled on AA men in the present study, however, CA men were selected and matched with AA men for major clinicopathological features (age, pathological grade,and outcome).
Blinding	The present study was performed in a blinded design (prospective-specimen-collection, retrospective-blinded-evaluation (PRoBE) design (Pepe et al, JNCI, 2008). The researchers and data analysts were blinded to the disease outcome, which was unblinded at data analysis.

Reporting for specific materials, systems and methods

We require information from authors about some types of materials, experimental systems and methods used in many studies. Here, indicate whether each material, system or method listed is relevant to your study. If you are not sure if a list item applies to your research, read the appropriate section before selecting a response.

Materials & experimental systems

n/a	Involved in the study
☒	☐ Antibodies
☒	☐ Eukaryotic cell lines
☒	☐ Palaeontology and archaeology
☒	☐ Animals and other organisms
☐	☐ Human research participants
☒	☐ Clinical data
☒	☐ Dual use research of concern

Methods

n/a	Involved in the study
☒	☐ ChIP-seq
☒	☐ Flow cytometry
☒	☐ MRI-based neuroimaging

Human research participants

Policy information about studies involving human research participants

Population characteristics	Dependent study outcomes include: Adverse pathology,Biochemical Recurrence, Distant Metastasis<br>Independent Study Predictors include:<br>Demographic and socio-demographic variables: Age at diagnosis, age at surgery, family history of CaP, self-reported patient race/ethnicity (African American, Caucasian), educational attainment, military rank.<br>Clinical and Pathological Characteristics: These data include: PSA at diagnosis, clinical stage, biopsy Gleason sum, pathological stage, pathological grade, seminal vesicle invasion, nodal status, extra-capsular extension (ECE) status, and surgical margin status.<br>Treatment Type: only patients who underwent radical prostatectomy (RP)—either open or robotic—were included since such patients are the source of biospecimens obtained at WRNMMC.<br>Risk Stratum: Patients in all NCCN risk strata which could be categorized into low, intermediate and high for formal comparisons.<br>Adjuvant and Salvage Therapy: Detailed information about the full scope of cancer treatments that a patient received was considered in the multivariable analysis. Given that the use of salvage therapy may influence cancer outcome, the timing of type of salvage therapy was included in the model.<br>Deployment status: A systematic medical chart review was conducted to ascertain the full history of patient deployments as part of their military career. The deployment location, deployment duration, and potential exposures per location were collected on each study subject.
Recruitment	Patients who provided informed consent to both the Center for Prostate Disease Research (CPDR) Multi-Center National Database and Biospecimen databank at the Walter Reed National Military Medical Center (WRNMMC) were the source of patients for this study. Eligible patients included all men aged 18 and older who were recommended for biopsy for detection of prostate cancer. Demographic, clinical, pathologic, treatment, and outcomes data were collected as part of routine patient follow up on all enrollees. Informed consent for the clinical database was obtained at the time of transrectal ultrasound guided biopsy (TRUS) for suspicion of CaP. Detailed patient demographic, clinical, treatment, and outcomes data were captured as part of routine data collection activities. All patients are military health care beneficiaries and their dependents.
Ethics oversight	Walter Reed National Military Medical Center; IRB Protocol # WRNMMC-2017-0122; Center for Prostate Disease Research (CPDR) Bio-Specimen Bank from Patients with Prostate Cancer, Benign Prostate Conditions, and Healthy Men" (WRNMMC IRB # 393738), Uniformed Services University Multi-Center National Database for the Center for Prostate Disease Research (CPDR) with Patterns of Care, Outcomes, and Prognostic Analyses" (WRNMMC # 385525)

Note that full information on the approval of the study protocol must also be provided in the manuscript.
